# Effect analysis of repeat sternotomy in pediatric cardiac operations

**DOI:** 10.1186/s13019-015-0381-z

**Published:** 2015-11-30

**Authors:** Chao-hua Yin, Jun Yan, Shou-jun Li, Dian-yuan Li, Qiang Wang, En-shi Wang

**Affiliations:** Department of Pediatric Cardiac Surgery, Cardiovascular Institute and Fuwai Hospital, Chinese Academy of Medical Sciences & Peking Union Medical College, Beijing, 100037 China

**Keywords:** Repeat sternotomy, Congenital heart disease, Major injury

## Abstract

**Background:**

Reoperation for congenital heart disease may be associated with cardiac or vascular injuries during repeat sternotomy, resulting in increased mortality and/or morbidity rates. The aim of this study was to determine the frequency of these cardiac injuries and the associated outcome.

**Methods:**

Between January 2012 and December 2013, 4256 sternotomy procedures were performed at the Pediatric Cardiac Center in Fuwai Hospital, including 195 repeat sternotomy procedures (RS). We retrospectively studied the clinical data of 195 RS patients and 250 randomly selected primary sternotomy (PS) patients. Demographic and operative details, major injures (MI), and clinical outcomes were compared between the two groups. We also assessed the risk factors for major injury and in-hospital mortality and morbidity.

**Results:**

Significant differences were observed between the RS and PS groups in terms of skin incision to cardiopulmonary bypass(CPB) time, overall CPB time, cross-clamp time and blood requirement, and ventilation time (*p* < 0.001). MI during RS occurred in 7 of the 195 patients (3.6 %), while operative mortality was 1.0 % (2/195). However, in the RS patients, mortality and morbidity rates were not significantly different between the MI subgroup and the non-MI subgroup (*p* = 1.000 and 0.556, respectively). Additionally, no significant difference was found between the RS and PS groups in terms of mortality (*p* = 1.000) and morbidity (*p* = 0.125).

**Conclusions:**

Both RS and MI are not associated with increased risk of operative mortality and morbidity. Outcomes for reoperative pediatric operations in contemporary practice are similar with those for primary operations.

## Background

Cardiac surgeries have greatly improved the survival rate of congenital heart disease (CHD) patients. During cardiac surgeries, sternotomy provides access to the heart and internal organs as a vertical incision is made along the sternum. However, with increasingly younger patients undergoing these surgeries, the need for repeat sternotomy (RS) to correct residual defects or new cardiac malformations have concurrently risen. RS are technically more difficult mainly due to the need to avoid injury of cardiac tissue and conduits implanted in the previous operation; therefore RS is considered as a significant risk of morbidity and mortality to the patient. However, there are only a few studies assessing RS in congenital heart surgery. DeLeon [1] and Russell [2] previously reported an about 5 % risk of injury to the heart or vascular structures during sternal reentry for procedures performed during the 1980s and 1990s. However, Morales [3] and Kirshbom [4] suggest that the risk of cardiac injury is less than 1 % and is not significantly different from primary sternotomy (PS). Herein, we reviewed the clinical data of 195 RS cases, assessing perioperative mortality and morbidity rates in comparison to a control group of PS patients. We compared the demographic, operative, and postoperative variables between these two groups, in order to improve the level of therapy for these patients.

## Methods

### Patients selection

The medical records of all RS (*n* = 195) procedures performed at the Pediatric Cardiac Center in Fuwai Hospital from January 2012 to December 2013 were retrospectively reviewed. During this same interval, 4256 median sternotomy procedures were performed; RS accounted for 4.6 % of all surgical procedures during this period. We randomly selected 250 patients retrospectively as the control group undergoing primary repair of a congenital heart defect during the same period. Demographic, operative, and postoperative variables were abstracted. RS was defined as a median sternotomy performed 30 days or longer after a previous median sternotomy [3]. This study was approved by the Institutional Review Board of Fu Wai Hospital.

### Surgical technique

External defibrillator and blood cell saver was used for all patients. Vein puncture needle was used to ensure quick fluid transfusion. Disinfection of chest, abdomen and bilateral groin was performed, and a marked line was drawn on the skin above the femoral artery. We used an oscillating saw to divide the anterior plate of the sternum, followed by division of the posterior plate of the sternum with electrocautery, then dissected the heart from the surrounding tissues and performed the cardiac surgery. The surgical procedures were completed by experienced cardiac surgeons.

### Statistical analysis

Normally distributed continuous variables of the RS and control PS groups were shown as mean ± standard deviation while other data were shown as median (range). Categorical variables were shown as frequency (percentage). The Kolmogorov-Smirnov test was used to determine whether the variables were normally distributed. Independent-sample *t* test or Mann–Whitney U test was used to compare the continuous variables between the two groups. Chi-square test, Fisher’s exact test or Mann–Whitney U-test was used to compare the non-continuous variables between the two groups. A *p*-value of less than 0.05 was considered statistically significant. All statistical analysis was performed using SPSS v.19 software.

## Results

### Perioperative characteristics

Overall, 4256 median sternotomy procedures were performed at the Pediatric Cardiac Center in Fuwai Hospital from January 2012 to December 2013, including 4061 (95.4 %) primary sternotomy (PS) and 195 (4.6 %) repeat sternotomy (RS). Of the RS patients, 188 (96.4 %) subjects had a history of one sternotomy, while 7 (3.6 %) had two previous sternotomy procedures.

Perioperative characteristics are summarized in Table 1. Compared with the randomly selected PS group, the patients’ age and body weight were significantly higher in the RS group (*p* <0.001). The median interval since the previous sternotomy was 35 (2–144) months in the RS group. Operative procedures performed after sternotomy of the RS and PS groups are listed in Table 2.

**Table 1:** Baseline characteristics of the repeat and primary sternotomy groups

Variable	RS (*n* = 195)	PS (*n* = 250)	*P*-value
Demographic			
Age (year)	5.2 (0.6–16.8)	1.1 (0.1–15.0)	0.000
Gender (female)	75 (38.5 %)	99 (39.6 %)	0.807
Weight (kg)	16.0 (5.0–55.0)	10.0 (3.3–47.0)	0.000
Interval since last sternotomy (month)	35 (2–144)	-	-
Operative			
Major injury	7 (3.6 %)	0	0.003
Incision-to-CPB time (min)	96 (35–271)	18 (15–37)	0.000
CPB time (min)	126 (0–442)	67 (0–206)	0.000
Cross-clamp time (min)	64 (0–222)	36 (0–153)	0.000
RBC requirement (units/kg)	0.06 (0–5.2)	0 (0–0.45)	0.000
Plasma requirement (mL/kg)	16.7 (0–260)	0 (0–64.5)	0.000
Chest tube drainage initial 24 h (mL/kg)	11.1 (2.7-76.4)	5.41 (2.5-18.5)	0.000
Ventilation time (h)	21 (1–1433)	9 (0–434)	0.000
ICU length of stay (d)	3 (0–65)	1 (0–35)	0.000
Outcomes			
Renal failure	6 (3.1 %)	7 (2.8 %)	0.863
Low output syndrome	5 (2.6 %)	6 (2.4 %)	1.000
Sepsis	5 (2.6 %)	3 (1.2 %)	0.306
Sternal infection/dehiscence	3 (1.5 %)	1 (0.4 %)	0.324
Bleeding	2 (1.0 %)	1 (0.4 %)	0.584
Total morbidity	21 (10.8 %)	18 (7.2 %)	0.125
Mortality	2 (1.0 %)	2 (0.8 %)	1.000

**Table 2:** Most common operative procedures performed after sternotomy

Procedure	RS (*n* = 195)	PS (*n* = 250)
Biventricular repair for cyanotic congenital heart disease^a^	62 (31.8 %)	45 (18.0 %)
Fontan procedure	57 (29.2 %)	5 (2.0 %)
Valve operation^b^	19 (9.7 %)	18 (7.2 %)
Switch^c^	15 (7.7 %)	9 (3.6 %)
Glenns	8 (4.1 %)	10 (4.0 %)
Left-to-right shunt^d^	7 (3.6 %)	122 (48.8 %)
Miscellaneous	27 (13.8 %)	41 (16.4 %)

^a^including tetralogy of Fallot, double outlet right ventricle and pulmonary artery atresia

^b^including valve repair and replacement, conduit replacement

^c^including arterial switch, double root translocation, and double switch surgery

^d^including atrial septal defect, ventricular septal defect, partial endocardial cushion defect, partial anomalous pulmonary venous drainage

### Operative characteristics

Major injury (MI) was defined as an injury resulting in non-elective peripheral cannulation for cardiopulmonary bypass, blood transfusion, orinitiation of inotropic drug treatment during management of the injury [3]. MI in RS group occurred in 7 of the 195 patients (3.6 %). The locations of the intraoperative injuries were the right atrium in four patients, the right ventricular injury in two patients, and an injury to the aorta in one patient. The femoral vessels were immediately explored and urgent femoral CPB was instituted. After that, the injured site was repaired and bleeding was controlled in all the 7 MI patients. Besides, elective femoral CPB was established before sternotomy in the two RS patients, and neither of them sustained MI.

The interval since last sternotomy in MI subgroup was 49(3–132) months, while 26(2–144) months in non-MI subgroup (*p* = 0.398). In the MI subgroup, all 7 RS patients had computer tomography (CT) or angiography preoperatively, and the pericardium had been closed in a previous operation in six of the subjects. Seven (4.5 %) of the 155 patients with preoperative imaging examination (including CT and angiography) sustained MI during RS, while none of the 40 patients without preoperative imaging examination had MI (0/40, 0 %, *p* =0.348).

The median skin incision to CPB time of the RS and PS groups were 96 (35–271) minutes and 18 (15–37) minutes, respectively (*p* <0.001). There was significant difference in CPB time (*p* <0.001) and cross-clamp time (*p* <0.001) between the two groups. Significant difference was also observed between the two groups in terms of blood requirement (*p* <0.001), chest tube drainage in initial 24 h (*p* <0.001), ventilation time (*p* <0.001), and ICU length of stay (*p* <0.001), as shown in Table 1.

In the RS group, pericardium closure during previous operations was reported in 147 patients. The incision-to-CPB time was 88(35–207) minutes in patients with pericardium closure (*n* = 89), while 98(52–271) minutes in those without closure (*n* = 58) (*p* = 0.207).

### Outcomes

Operative mortality was 1.0 % (2/195) in the RS group and 0.8 % (2/250) in the PS group, with no significant difference between the two groups (*p* = 1.000). The causes of death in RS group included sepsis shock in one patient and low cardiac output syndrome in a second patient. The overall mortality of primary sternotomy in the same period was 0.9 % (38/4061), with no significance observed as compared to the RS group (*p* = 0.9). In the RS patients, mortality was not significant between the MI subgroup (0/7, 0 %) and the non-MI subgroup (2/188, 1.1 %) (*p* = 1.000), also between first-time RS (2/188, 1.1 %) and second-time RS (0/7, 0 %; *p* = 1.000). In addition, the total morbidity of the MI and non-MI subgroup was 14.3 % (1/7) and 10.6 % (20/188), without significant difference (*p* = 0.556).

The most common morbidities in the RS group were renal failure (6/195, 3.1 %), low output syndrome (5/195, 2.6 %), and sepsis (5/195, 2.6 %). Sternal wound infection or dehiscence occurred in 1.54 % (3/195) of patients. Postoperative bleeding requiring mediastinal exploration occurred in 1.0 % (2/195) of RS patients. We compared these main complications and no significant difference was found between the RS and PS groups, as shown in Table 1. On regression analysis, neither operative mortality (*p* = 0.803) nor morbidity (*p* = 0.138) were significantly correlated with RS.

## Discussion

As operative techniques evolve and survival rates improve after pediatric cardiac operations, the number of CHD patients receiving RS inevitably continues to rise. Herein, we summarized the RS procedures in comparison to PS at Fuwai Hospital in China. The most common operative procedures performed after RS was biverntricular repair for cyanotic congential heart disease, which is different from previous reports [3, 4]. The occurrence of MI in our study was 3.6 %, which was similar to the previously published literature [1–4] of 1–5 %. There was no statistical difference in operative mortality and morbidity between the MI group and non-MI, which was consistent with a report from Kirshbom [4] that showed that MI does not increase operative mortality and morbidity. Our operative mortality in the RS group compared with the control PS group showed no statistical difference (1 % *versus* 0.8 %*, p =* 1.0*),* which was consistent with Morales’ [3] report that showed RS was not a risk factor of pediatric cardiac operative mortality. The Kirshbom study [4] reported that mortality was associated with the number of sternotomy procedures, while the Swartz study [5] reported that increasing number of previous sternotomies does not increase the overall morbidity and mortality in some patients. Our data also showed the mortality was not associated with the number of sternotomy procedures, but we only had 7 s RS patients. In summary, both our data and published literature showed that RS was not a risk factor for CHD and the occurrence of MI did not increase the operative mortality and morbidity.

Our method of RS was mainly as follows. The previously placed sternal wires were divided but not removed. The sternum was divided using an oscillating saw down to the sternal wires which represented the beginning of the posterior plate of the sternum. The sternal wires were then removed. The sternum was retracted upward and laterally, the posterior plate beneath which there were no important structures that could be easily injured, and were therefore incised with the electrocautery. This allowed the visualization of meticulous structures such as the aorta, and then all of the remaining posterior plate was carefully incised. Importantly, freeing the heart from the surrounding tissue required finding the gap between the heart and pericardium; therefore knowledge of the cardiac structure was critical. Preoperative image data and previous operative reports were also necessary for patient evaluation. Electrocautery should be performed quickly to avoid arrhythmia or ventricular fibrillation [6]. During sternotomy surgery, anesthesia and resuscitation teams should be ready to perform emergency CPB. Once hemorrhage occurs especially during division of the sternum, the sternotomy should be discontinued immediately. Additionally, the femoral CPB should be established rapidly and MI must be repaired when the core temperature and the flow reduces. Athanasiou [7] reported application of endoscopy for RS also achieved satisfactory results.

The procedure of the primary operation is important for the outcome of RS. Dobell [8] reported the pericardium had not been closed in 88 % of MI patients in the previous operation. Boyd [9] considered that the pericardium should be closed, so as to avoid the adhesion of epicardial and sternum, thus reduce the occurrence of MI. Our data showed that the time from skin incision to establish CPB in patients whose pericardium had been closed previously was shorter than not, even though these differences were not statistically significant. To close the pericardium might be helpful for RS and the subsequent freeing of the heart and great vessels from surrounding tissues. We avoided removing all the thymus and sealed the pericardium as often as possible in patients undergoing staged repairs. The artificial vascular slice was considered when the pericardium was inadequate for closure. We also avoided using bovine pericardium because it adhered to the epicardium thus it was difficult to dissect. Napoleone [10] found that the adhesion barrier film was considered ideal for prevention of cardiac adhesions. The interval between operation may also influence the occurrence of MI. Park [11] reported that patients whose previous operations had been performed less than 12 months prior had a greater incidence of injury during RS. However, Lodge [12] reported that the interval between the two surgeries was of no consequence regardless of the degree of cardiac adhesion, as the interval in MI group showed no difference with others. Our data suggested that the interval did not appear statistically different between the MI group and the non-MI group, the mean interval in the two groups were all more than 2 years.

Both Morales [3] and Kirshbom [4] reported that preoperative imaging examination was not necessary, but Hamid [13] showed that preoperative CT scans were helpful to reduce MI. To clearly understand cardiac structure and pulmonary vascular development, the majority of our patients (155/195, 79.5 %) underwent CT or angiography, which also provided the adhesive conditions of retrosternal tissue. However, the MI patients had all imaging examination (7/155, 4.5 %) while the patients without imaging examination did not suffer MI (0/40, 0 %, *p* =0.348), this may be attributed to different cardiac malformations of patients and confounded by the selection bias inherent by the patients who underwent preoperative imaging examination as high risk. Multiple reoperation and conduit was a risk factor in patients with MI occurrence [4]. Even if the echocardiography could determine the operation plan, CT examination was also required to clear the adhesive conditions of retrosternal structure in this subgroup. In special conditions, such as reported by Herman [14], the pseudoaneurysm and erosion were visualized with CT angiography before RS. The patient was placed on cardiopulmonary bypass *via* the femoral vessels before median sternotomy, injury occurred as expected, but there was only minimal bleeding. The operation was completed successfully and the patient recovered well. This case report highlights the importance of preoperative imaging in special patients.

It is somewhat difficult to determine the effectiveness of elective femoral CPB. Luciani [15] reported that this method can make re-operation safer and simpler in adults. However, our patients were mainly children and infants. Thin and small femoral artery of this patient cohort therefore makes cannulation difficult, and the patient is more prone to vascular complications, and it is difficult to achieve full-flow CPB. Meanwhile, bleeding after heparinization makes it more difficult to free the heart from the surrounding tissue. Our strategy was to therefore avoid this method. In high risk patients, femoral artery puncture needle may be kept for young children with low weight; while the femoral vessel may be kept exposed for older children with high weight. Once needed, we could quickly establish femoral CPB. Even in the cases that required the urgent establishment of femoral CPB to control hemorrhage, we also performed main cardiac surgery by conventional extracorporeal circulation with cannulation of both *vena cava* and the ascending aorta after controlling bleeding. We had some cases of femoral CPB which appeared as lower limb ischemia(not in this study). After we routinely change the location of arterial cannulation from the femoral artery to the ascending aorta, lower limb ischemia never happened. Kirshbom [4] reported that patients did not benefit from femoral CPB, while Ellmanetc [16] reported that it did not reduce the incidence of MI. However, in a special patient, femoral CPB was necessary as reported by Herman [14]. Only two patients in our study used femoral CPB before RS, and they did not suffer subsequent MI. Therefore, additional studies are needed to determine the effectiveness of CPB.

Our study has important limitations. As with any retrospective study, data collection was subject to attribution error. The description of the MI comes from the surgical records; therefore some MI may not have been recorded.

## Conclusions

In summary, according to our clinical data, although major injury was observed during repeat sternotomy, neither MI and RS are associated with increased risk of operative mortality and morbidity. Preoperative imaging examination maybe necessary in high risk patients. We routinely change the location of arterial cannulation from the femoral artery to the ascending aorta in order to avoid complications of lower limb ischemia if we use femoral CPB. With careful planning and execution, outcomes for reoperative pediatric operations in contemporary practice are similar with those for primary operations. RS in pediatric cardiac operations is therefore a relatively safe procedure and the choice of clinical management strategies for a patient should not be affected by the need for RS.

## Abbreviations

RS: Repeat sternotomy
MI: Major injuries
CPB: Cardiopulmonary bypass
CHD: Congenital heart disease
